# Distributed Airflow Sensing Based on High-Spatial-Resolution BOTDA and a Self-Heated High-Attenuation Fiber

**DOI:** 10.3390/s22114017

**Published:** 2022-05-26

**Authors:** Hongying Zhang, Yanyang Lei, Jinzhe Zhou, Yongkang Dong

**Affiliations:** 1 Heilongjiang Provincial Key Laboratory of Quantum Control, School of Measurement and Communication Engineering, Harbin University of Science and Technology, Harbin 150080, China; zhy_hit@163.com (H.Z.); 1920910087@stu.hrbust.edu.cn (Y.L.); 2 School of Science, Harbin University of Science and Technology, Harbin 150080, China; zjz18845725313@126.com; 3 National Key Laboratory of Science and Technology on Tunable Laser, Harbin Institute of Technology, Harbin 150001, China

**Keywords:** distributed airflow sensing, Brillouin optical time domain analysis, high-attenuation fiber, Brillouin frequency shift

## Abstract

An all-fiber distributed airflow sensing method based on a differential pulse-width pair Brillouin optical time domain analysis (DPP-BOTDA) and a self-heated high-attenuation fiber (HAF) is proposed and demonstrated. The HAF heated the sensing fiber, producing a gradient temperature distribution in it through physical contact, where the temperature distribution was obtained by DPP-BOTDA with a spatial resolution of 5 cm. The heat loss caused by the airflow was reflected in the decrease in the Brillouin frequency shift and spatially resolved by DPP-BOTDA. Distributed airflow sensing was experimentally demonstrated for measurements of airflow movement, multiple airflow sources and the deflection angle of the airflow. The positioning error of the airflow was no larger than ~2.2 cm; for the deflection angle measurements of the airflow, the maximum demodulation error was 2.5° within the angle range of 0–30°.

## 1. Introduction

The measurements of gas and liquid flow are necessary in many important fields related to the national economy and the people’s livelihoods, such as industrial production, environmental monitoring, detection of gas/liquid pipe leaks and so on [1]. A typical design for flow sensing is a hot wire anemometer (HWA), whose principle is to measure the heat taken away by the blowing gas/liquid from the hot wires/films. Optical fiber sensors have attracted more and more attention because of their advantages of fast response, compact structure, inherent safety, anti-electromagnetic interference, harsh environment adaptability and so on.

As an ideal alternative to traditional electronics or microelectromechanical systems-based flow sensors under harsh environments, many flow sensors based on optical fiber sensing technology have been studied and reported over the past two decades. Different types of fibers and thermal coupling structures were used for designs of optical fiber-based flow sensors with hot wire measuring principles, such as single-mode–multimode junction [2,3], high-attenuation fiber (HAF) [4], no-core fiber [5], long-period grating/fiber Bragg grating (FBG) structure [6], core-offset fiber structure [7,8], fiber taper structure [9,10,11], etc. Among these studies, various types of FBGs were mostly used as the sensing element for indirect flow information acquisitions through measurements of reflection spectrum shift. Making use of the high-temperature characteristics of the regenerated fiber Bragg grating, flow sensing with temperatures even up to 800 °C was achieved by Rongzhang Chen et al., based on self-heated HAFs [12]. In 2017, Yang Zhang et al. proposed a low-power-consumption, all-fiber-optic anemometer based on a tilted fiber Bragg grating that was coated with single-walled carbon nanotubes [13]. More recently, a hot-wire flowmeter, using a fiber extrinsic Fabry–Pérot interferometer combined with a FBG, was reported by Tianxi Zhang et al. [14].

The linear structure of an optical fiber makes it an ideal carrier for distributed sensing; however, there are limitations for grating-based point optical fiber flow sensors for applications that require distributed measurements. By building a long piece of self-heated optical fiber into a multi-layer fiber hotwire grid, Tong Chen et al. presented distributed flow sensing in a millimeter resolution using optical frequency domain reflectometry [4]. Moreover, to the best of our knowledge, there are few other distributed flow sensors that have been reported. Therefore, distributed flow sensing techniques still remain to be developed further.

Distributed Brillouin optical fiber sensing technology has attracted increasing attention because of its high performance over long distances, high spatial resolutions, fast measurements and multi parameter measurements, etc. In order to improve its spatial resolution, several methods have been developed over the past decade, such as pulse pre-pump technique, differential pulse-width pair (DPP) technique and so on for the Brillouin optical time domain analysis (BOTDA) [15].

In this paper, we proposed and demonstrated a novel type of all-fiber and distributed airflow sensing method based on DPP-BOTDA and a self-heated HAF. The sensor consisted of a common single-mode fiber (SMF) as the sensing fiber and a self-heated HAF that was glued alongside the SMF, so that the SMF could be heated through physical contact to become a “hot wire”. Compared with the distributed optical fiber flow sensing method in [4], which used a single HAF as both the sensing fiber and the heating fiber, these two fibers are separate in our method, with the sensing fiber offering a very low loss. Therefore, a longer measurement length can be achieved, though the accuracy will be reduced to some extent because of the tradeoff between them, which is the advantage of our method. Through temperature distribution measurements of the heated sensing fiber using DPP-BOTDA with a spatial resolution of 5 cm, information such as the position and direction of the airflow can be obtained by using only fibers. Measurements of a single airflow, multiple airflows and the deflection angle of the airflow were demonstrated using the proposed method, providing a new flow-sensing method based on distributed optical fiber-sensing technology.

## 2. Experimental Setup and Measurement Principle

**Figure 1:**
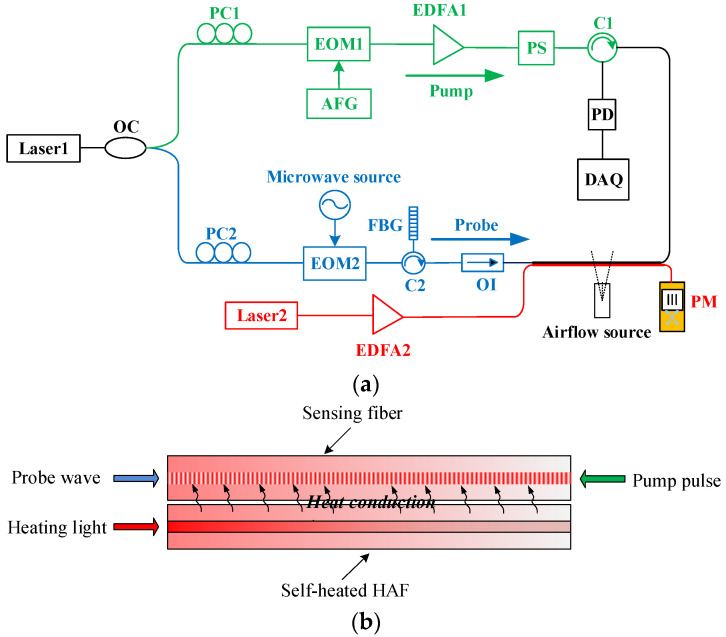
(**a**) The experimental setup and (**b**) schematic diagram of the fiber layout. OC: Optical coupler; PC: Polarization controller; EOM: Electro-optic modulator; AFG: Arbitrary function generator; EDFA: Erbium-doped fiber amplifier; PS: Polarization scrambler; C: Circulator; PD: Photodetector; DAQ: Data acquisition card; FBG: Fiber Bragg grating; OI: Optical isolator; PM: Power meter.

The experimental setup we used for airflow sensing consisted of the classical scheme of DPP-BOTDA [16,17] and the heating structure of physical heat conduction using a HAF, as shown in Figure 1. A narrow-band fiber laser (laser1) operating at 1550 nm was adopted as the light source, whose output was split into two paths by an optical coupler (OC) to provide the pump and the probe waves, respectively. The light in one optical path (green) entered a high-extinction-ratio electro-optic modulator (EOM1) that was driven by an arbitrary function generator (AFG) to be modulated into the pump pulse with an extinction ratio higher than 40 dB. A pulse pair of 8/8.5 ns was used to provide a spatial resolution of 5 cm. After being amplified by an Erbium doped fiber amplifier (EDFA1), the pump pulse pair finally entered the sensing fiber, which was a 5 m standard SMF, and the polarization scrambler (PS) in front of it was used to avoid fluctuation of the polarization-dependent Brillouin signal.

In the other path (blue), the light was modulated by the EOM2 that was driven by a microwave source, resulting in a signal with ±1 order sidebands. The frequency difference between the sidebands and the carrier wave was equal to the microwave frequency, i.e., the Brillouin frequency shift (BFS) of the sensing fiber. After filtering by a FBG with a 3 dB bandwidth of 0.03 nm, the first-order lower sideband was selected as the probe wave. The probe wave passed through an optical isolator (OI), and then entered the sensing optical fiber, where it was amplified by the pump pulse through the interaction of stimulated Brillouin scattering. The main purpose of using the OI was to prevent the high-power pump light from passing back through the probe path and damaging the devices and laser source. The amplified probe light was converted into an electrical signal by the photodetector (PD) and, finally, collected by the data acquisition card (DAQ) with a sampling rate of 10 GHz/s and a sampling resolution of 1 cm. In the experiment, the data were averaged 5000 times.

To heat the sensing fiber, a self-heated HAF (red) was used as the heating fiber. The layout of the two fibers is shown in Figure 1b, where they are arranged alongside and adhered to each other using an epoxy resin glue to form good physical contact between them. The 1550 nm continuous-wave output from the laser was amplified by the EDFA2 and then entered the HAF, with a power meter (PM) monitoring its input optical power. The self-heated HAF is a type of fiber doped with cobalt or germanium in the core region, and the nonradiative absorption of the incident light by the doped ions produced a thermal effect, i.e., efficiently converted the laser energy into heat energy, resulting in a significant increase in the temperature of the fiber, with a temperature rise of $\Delta T_{s}$, which is almost uniform across the fiber cross section at a steady-state under continuous pumping, approximated by [18]:

(1) $$\Delta T_{s}(z)≐-\frac{\eta }{2\pi Rh}\cdot \frac{dP_{H}(z)}{dz}$$

where *z* is the position along the fiber, η is the fraction of light energy absorbed per unit volume, *R* is the outer radius of the fiber, *h* is the heat transfer coefficient, and $P_{H}(z)$ is the power distribution of the heating light along the fiber.

The light power will decay because of the absorption loss as it propagates along the HAF, with the expression of:

(2) $$P_{H}(z)=P_{0}\cdot \exp(-\alpha z)$$

where $P_{0}$ and α are the incident light power and the attenuation coefficient of the HAF, respectively. By combining Equations (1) and (2), a temperature distribution with an approximately exponential attenuation could be formed along the HAF.

Subsequently, the sensing fiber could be heated through heat conduction, obtaining a similar distribution of temperature rise with a scale coefficient κ, which was assumed to be uniform along the fiber. There is a linear relationship between the BFS and the temperature change in the fiber, i.e., $\Delta \upsilon_{B}=C_{B}^{T}\Delta T$, where $\Delta \upsilon_{B}$ and ΔT are changes in the BFS and the temperature of the sensing fiber, respectively, and $C_{B}^{T}$ is the temperature coefficient of the BFS. Therefore, a similar shape of BFS distribution will finally be formed in the sensing fiber, and can be simply expressed by:

(3) $$\Delta \upsilon_{B}(z)=\xi \cdot \exp(-\alpha z)$$

where ξ is the total coefficient taking the form of:

(4) $$\xi =\frac{\alpha \eta \kappa }{2\pi Rh}C_{B}^{T}$$

Equation (3) clearly indicates that the BFS increase, with a distribution of approximately exponential attenuation, will finally be formed in the sensing fiber. When the sensing fiber is blown by the airflow, the heat at the blown position will be taken away, resulting in a local temperature drop and, correspondingly, a decrease in the BFS because of its linear dependence on temperature. Therefore, the airflow can be detected and localized by the distributed measurement of the fiber BFS.

## 3. Results and Discussions

### 3.1. Characterization of the Sensing Head

**Figure 2:**
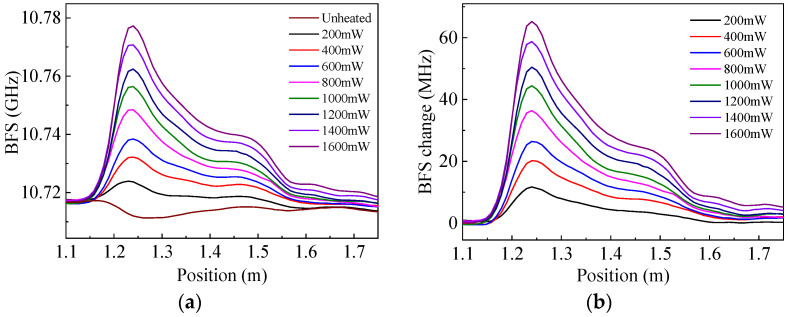
Brillouin frequency shift (BFS) distributions of the sensing optical fiber under different heating powers of the self-heated high-attenuation fiber (HAF): (**a**) Distributions of BFS; (**b**) Distributions of BFS change.

In order to characterize the BFS distributions of the heated sensing fiber, the HAF was heated by different laser powers from 200 to 1600 mW. The measured BFS distributions of the sensing fiber is shown in Figure 2a, where the curve for unheated fiber represents the original BFS distribution without heating. In the experiment, the section of 1.15–2.15 m on the sensing fiber was glued alongside the HAF, whose attenuation coefficient and length were 20 dB/m and 1 m, respectively. It can be seen from the figure that the BFS of the heated sensing fiber section increases as the injected optical power of the HAF increases, with the maximum BFS appearing at the position of 1.24 m; this also reflects the temperature distributions because of the linear temperature dependence of the BFS.

The measured BFS distributions under heating were subtracted by the unheated distribution to obtain the BFS changes, as shown in Figure 2b. The results show that the higher the heating power of the HAF, the greater the BFS increase in the sensing fiber, and the maximum increase was at the position of 1.24 m. The maximum BFS increase corresponds to the highest temperature, which is expected to be located at the position of the highest heating power of the HAF (1.15 m of the sensing fiber). However, this location is shifted backward by a short distance (about 10 cm) in our measurement results, which may be caused by some optical power loss due to the higher thermal conductivity of silica compared to that of the cladding at the fiber connection; another possible reason for this displacement may be the nonuniformity of glue application.

**Figure 3:**
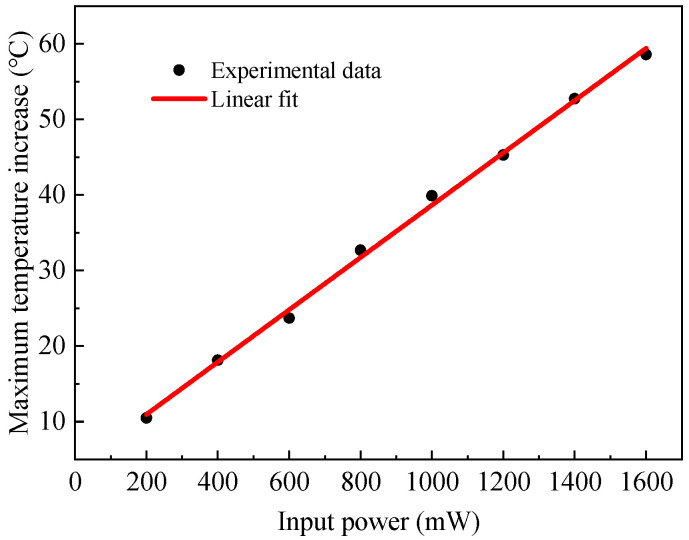
The maximum temperature change in the sensing fiber vs. input power of the HAF.

Using the BFS temperature coefficient of 1.113 MHz/°C for single-mode fibers [19], the relationship between the maximum temperature increase in the sensing fiber and heating power of the HAF was obtained, as shown in Figure 3. The black dots in the figure are the experimental results, and the red line is the linear fit. It can be seen that the maximum temperature change increases with the increase in the heating power, and there is a good linear relationship between them. The maximum temperature increase is as high as 58.6 °C with a heating power of 1600 mW. It is believed that higher temperature increases can be obtained by optimizing the heating structure, such as using a glue with a better thermal conductivity or removing the cladding of the HAF so that it can directly contact the sensing fiber for more efficient heat transfer.

### 3.2. Measurement of Transverse Movement of the Airflow

**Figure 4:**
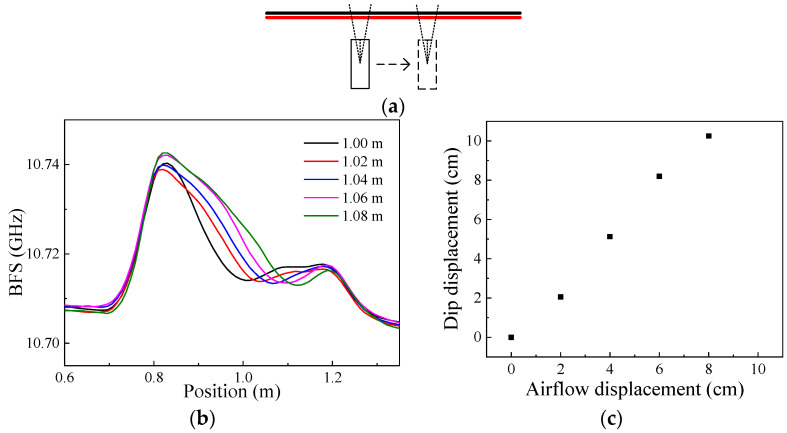
Schematic diagram and measurement results for transverse movement of the airflow: (**a**) Schematic diagram for transverse movement of the airflow; (**b**) BFS distributions for different airflow positions; (**c**) The demodulation result.

The transverse movement of the airflow was measured first. Keeping the vertical distance between the airflow source and the sensing fiber unchanged (~13 cm), the airflow source was continuously moved with a step of 2 cm in the direction parallel to the sensing fiber. A schematic diagram is shown in Figure 4a, where the black and red lines are the sensing fiber and the HAF, respectively. Under an 800 mW heating optical power of the HAF, the BFS distributions of the sensing fiber were measured when the airflow source was at horizontal positions of 1, 1.02, 1.04, 1.06 and 1.08 m, respectively; the results are shown in Figure 4b. It can be seen from the figure that the BFS of the sensing fiber decreases to form a dip in the curve, which is because of the heat loss caused by the blowing airflow, and the dip moves accordingly with the position of the airflow source.

The measured dip displacement vs. the actual displacement of the airflow is shown in Figure 4c, and the maximum deviation of ~2.2 cm can be obtained within the measurement range, which is mainly limited by the spatial resolution of the system. It should be noted that under the heating scheme in this paper, the measurement range for airflow displacement was limited by the effective sensing length, i.e., the fiber length corresponding to the falling edge of the BFS curve, which is expected to be improved in further studies by improving the heating structure.

### 3.3. Measurement of Multiple Airflows

**Figure 5:**
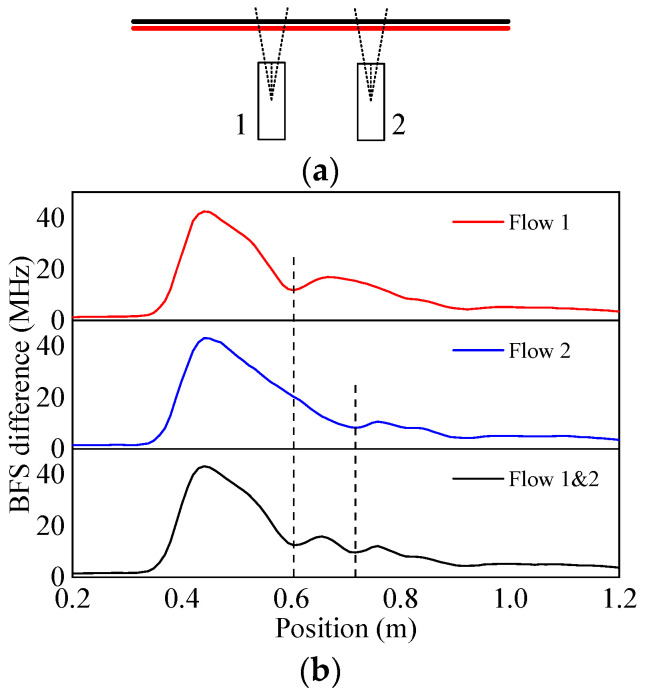
Schematic diagram and measurement results for two airflows blowing simultaneously: (**a**) Schematic diagram of two airflows blowing simultaneously; (**b**) Distributions of BFS difference when the sensing fiber was blown by airflow 1 (red), 2 (blue) and both (black).

Based on the above results, the scenario of two airflows was also studied. A diagram is shown in Figure 5a, where the two airflow sources are denoted by 1 and 2, respectively. The distance between their centers was about 10 cm, and each airflow generated by them could be turned on or off by separately controlling their switches. In this experiment, the heating optical power of the HAF was 800 mW. The measured distributions of the BFS difference are shown in Figure 5b, where the red and blue curves are the results when airflow 1 and 2 blow alone, respectively, while the black curve is the result when they are both blowing. Both curves for airflows 1 and 2 each have one dip at the positions of 0.604 m and 0.717 m, respectively, indicating that the two airflows blow separately. Two heat loss dips can be distinguished at these two positions on the black curve, indicating two airflows blowing at the same time and a positioning accuracy of 1.3 cm compared to the actual distance between them. The experimental results indicate the capability of the distributed sensing of multiple airflow sources of our method.

### 3.4. Measurement of Deflection Angle of the Airflow

**Figure 6:**
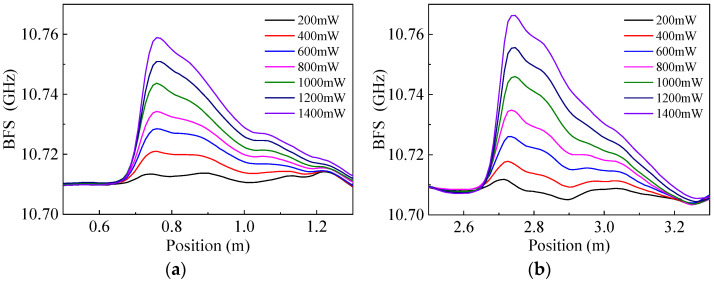
BFS distributions of the two heated sections of the sensing fiber at different input optical powers of the HAFs: (**a**) Section 1; (**b**) Section 2.

Further experiments were implemented to verify the ability to measure the deflection angle of the airflow by using the method proposed in this paper. Two sensing fiber sections were required in order to obtain the information of deflection angle; therefore, the BFS distributions of the sensing fiber with two separate sections heated at the same time were measured first. Two HAFs with a length of 1 m were separated by a short distance apart and glued alongside the sensing optical fiber, respectively. The BFS distributions of the sensing fiber when the two HAFs were heated by laser powers of 200–1400 mW are shown in Figure 6, where (a) and (b) are those of the sections 1 and 2, respectively. It can be seen from the figures that the BFS distributions of the two heated sections have the same characteristics as those shown in Figure 2a, that is, the higher the heating power, the greater the BFS increase.

**Figure 7:**
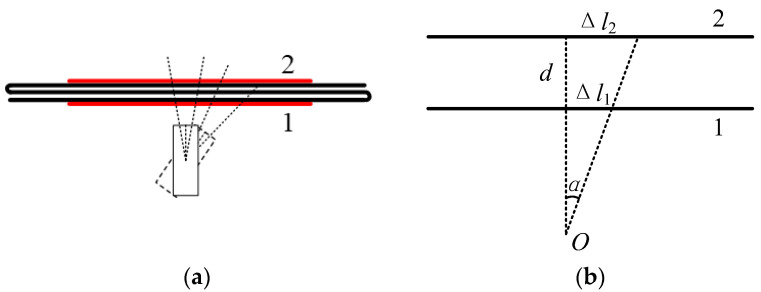
Schematic diagram of the airflow deflection and the corresponding geometric relationship of the deflection angle: (**a**) Diagram of the deflection angle measurement; (**b**) Geometric relationship of the deflection angle.

The sensing fiber was S-shaped and arranged so that the two heated sections could be in parallel with a vertical spacing of ~8 cm, as shown in Figure 7a, where the two fiber sections close to and far from the airflow source are marked as 1 and 2, respectively. The airflow source was fixed with the vertical distance of about 13 cm from the fiber section 1, and generated the airflow at different angles when it was deflected. The demodulation principle of the deflection angle is shown in Figure 7b, where *O* is the rotation center of the airflow source, *d* is the vertical distance between the two heated sections of the sensing fiber, Δ*l*_1_ and Δ*l*_2_ are the displacements of the blown locations on fiber sections 1 and 2, respectively, and *α* is the deflection angle of the airflow, which can be calculated by the following Equation [4]:

*α* = arctan[(Δ*l*_2_ − Δ*l*_1_)/*d*] (5)

**Figure 8:**
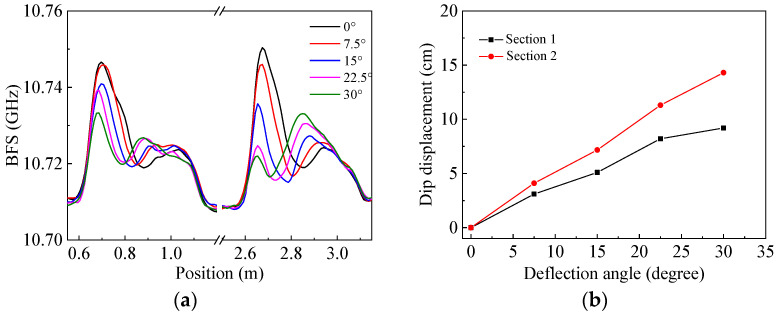
Measurement results of airflow direction: (**a**) BFS distributions of the sensing fiber at different deflection angles of the airflow; (**b**) Dip displacement vs. deflection angle for fiber sections 1 (black) and 2 (red), respectively.

By rotating the airflow source from the vertical blowing direction (0°) to deflection angles of 7.5°, 15°, 22.5° and 30°, the corresponding BFS distributions of the sensing fiber were measured, as shown in Figure 8a, where the regions of 0.6–1.2 m and 2.5–3.1 m correspond to the fiber sections 1 and 2 in Figure 7, respectively. It can be clearly seen from the figure that the initial dip positions corresponding to the deflection angle of 0° are at 0.881 and 2.849 m for fiber sections 1 and 2, respectively, and they both move away from their initial locations to the smaller value side regularly. In other words, the dip displacements both become larger, relative to their respective initial positions, as the deflection angle increases from 0° to 7.5°, 15°, 22.5° and 30°, respectively, indicating that the blown locations on the two fiber sections move in the same direction as the deflection angle increases. Figure 8b shows the dependence of the dip displacements on the deflection angle, where the black squares and red dots are those for fiber sections 1 and 2, respectively. The displacement of the dip position for section 2 is around 1.4 times that for section 1 at the same deflection angle, which is caused by the different distances between the airflow source and each fiber section.

Based on the measurement results shown in Figure 8b, the deflection angles of the airflow can be demodulated by using Equation (5), with the results shown in Table 1, where the actual deflection angles are also listed for the convenience of comparison. The small deviations between them are mainly caused by the certain cross section size of the airflow that leads to the inaccurate location of the heat loss dips. The deviation increases with the increase in the deflection angle, with the minimum and maximum values being 0.375° and −2.5° at the actual angles of 7.5° and 30°, respectively, which is because of the greater distance between the blown location on the sensing fiber and the airflow source at a larger deflection angle.

It should be mentioned that the airflow used in our experiment was a slightly divergent shape, resulting in a certain width on the sensing fiber blown by the airflow. Theoretically speaking, by increasing distances between the airflow source and the sensing optical fiber, as well as that between the two heated fiber sections, the measurement accuracy and capability of demodulating relatively small angle changes will be improved because the displacement of the blown location becomes larger under the same deflection angle. Furthermore, on the one hand, the size of the blown region on the sensing fiber will also become larger, making it more difficult to accurately determine the dip position; on the other hand, the dips will become more inconspicuous because of the relatively weaker airflow, reducing the effective measurement range of the deflection angle. The distances used in our experiment were carefully selected under existing conditions, and we believe that the measurement accuracy can be effectively improved by using a better airflow source to generate airflows with a better directivity and smaller divergence angle.

## 4. Conclusions

In conclusion, we proposed and experimentally demonstrated an all-optical fiber distributed airflow sensing method based on DPP-BOTDA with the assistance of a self-heated HAF in this paper. The temperature decrease induced by the airflow was detected by the interrogation of the BFS distribution through the technique of DPP-BOTDA. Measurements of single and multiple airflows, and a deflection angle of the airflow, were experimentally demonstrated, with the results indicating the capability of distributed airflow sensing using our method. The airflow displacement was measured, and two airflows blowing simultaneously were distinguished, with the maximum positioning error of ~2.2 cm. Within the measured deflection angle range of 0–30°, the maximum deviation between the demodulated and actual angles was 2.5°. Better performances can be expected by using improved heating structure and airflow sources, which need to be investigated in the future work. Taking the advantages of distributed optical fiber sensing technology, the proposed method is expected to be a potential candidate for harsh environments and distributed flow sensing applications.

## Figures and Tables

**Table 1:** The demodulated and actual deflection angles of the airflow (°).

Demodulated angle	7.125	14.5	21.18	32.5
Actual angle	7.5	15	22.5	30
Deviation	0.375	0.5	1.32	−2.5

## Data Availability

Not applicable.
